# Recording of Social Determinants in Computerized Medical Records in Primary Care Consultations: Quasi-Experimental Study

**DOI:** 10.2196/41706

**Published:** 2023-01-25

**Authors:** Berta Rodoreda-Pallàs, Iris Lumillo-Gutiérrez, Queralt Miró Catalina, Eva Torra Escarrer, Jaume Sanahuja Juncadella, Victoria Morin Fraile

**Affiliations:** 1 Santpedor Primary Health Care, EAP Navarcles/Sant Frutiós /Santpedor, Primary Care Service Bages-Berguedà Central Catalonia Territorial Management Institut Català de la Salut Santpedor Spain; 2 Health Promotion in Rural Areas Research Group Institut Català de la Salut Sant Fruitós de Bages Spain; 3 Research Support Unit of Central Catalonia Fundació Institut Universitari per a la Recerca a l'Atenció Primària de Salut Jordi Gol i Gurina Barcelona Spain; 4 Department of Public Health, Mental Health and Maternal and Child Health Nursing Universitat de Barcelona Barcelona Spain; 5 Chronicity and Complexity Care Unit, Baix Llobregat Centre Primary Care Service Southern Metropolitan Territorial Management Institut Català de la Salut Cornellà de Llobregat (Barcelona) Spain; 6 Research Group on Environments and Materials for Learning Universitat de Barcelona Barcelona Spain; 7 Sant Vicenç de Castellet Primary Health Care, Primary Care Service Bages-Berguedà Central Catalonia Territorial Management Institut Català de la Salut Sant Vicenç de Castellet Spain; 8 Plaça Catalunya Primary Health Care, Primary Care Service Bages-Berguedà Central Catalonia Territorial Management Institut Català de la Salut Manresa Spain; 9 Health Education and Promotion Universitat de Barcelona Barcelona Spain; 10 School of Nursing Universitat de Barcelona Barcelona Spain

**Keywords:** recording of social determinants of health, computerised medical records, electronic health record coding, non-clinical diagnoses, Z-coding, primary care, medical records, intervention, medical, treatment

## Abstract

**Background:**

Social determinants of health may be more important than medical or lifestyle choices in influencing people's health. Even so, there is a deficit in recording these in patients' computerized medical histories. The Spanish administration and the World Health Organization are promoting the recording of diagnoses in computerized clinical histories with the aim of benefiting the individual, the professional, and the community. In most cases, professionals tend to record only clinical diagnoses despite evidence in the literature documenting that addressing the social determinants of health can lead to improvements in health and reductions in social disparities in disease.

**Objective:**

This study aims to develop and evaluate the effectiveness of a mixed intervention (face-to-face-digital) aimed at improving the quantity and quality of the records of the social determinants of health in computerized medical records at primary care clinics.

**Methods:**

A quasi-experimental, nonrandomized, controlled, multicenter study with 2 parallel study arms was conducted in the area of Central Catalonia (Spain) with primary care professionals of the Institut Català de la Salut (ICS), working from September 23, 2019, to March 31, 2020. All interested professionals were accepted. In total, 22 basic health areas were involved in the study. In Spain and Catalonia, the International Classification of Diseases is used, in which there is a coding of the social determinants of health. Five social determinants were selected by a physician, a nurse, and a social worker; these professionals had experience in primary care and were experts in community health. The choice was made taking into account the ease of use, benefit, and existing terminology. The intervention, based on the integration of a checklist, was integrated as part of the usual multidisciplinary clinical workflow in primary care consultations to influence the recording of these determinants in the patient's computerized medical record.

**Results:**

After 6 months of implementing the intervention, the volume and quantity of records of 5 social determinants of health were compared, and a significant increase in the median number of pre- and postintervention diagnoses was observed (*P*≤.001). There was also an increase in the diversity of selected social determinants. Using the linear regression model, the significant mean increase of the experimental group with respect to the control group was estimated with a coefficient of 8.18 (95% CI 5.11-11.26).

**Conclusions:**

The intervention described in this study is an effective tool for coding the social determinants of health designed by a multidisciplinary team to be incorporated into the workflow of primary care practices. The effectiveness of its usability and the description of the intervention described here should be generalizable to any environment.

**Trial Registration:**

ClinicalTrials.gov NCT04151056; https://clinicaltrials.gov/ct2/show/NCT04151056

## Introduction

Health depends on the circumstances in which people are born, grow up, live, work, and age, including the health care system [1]. These circumstances, considered social determinants of health (SDHs), are described and analyzed by various models and theories [2].

Among them, it is worth highlighting the framework proposed by Solar and Irwin from the WHO Equity Team. This framework proposed by the WHO Equity Team at the World Health Organization (WHO) contains 2 main elements: structural factors and intermediate factors of health inequalities. The structural ones are composed of the socioeconomic, political, and social structure context. The intermediate factors are composed of the material resources that in turn influence the psychosocial processes and the health-influencing behaviors and biological processes that derive from them and the ending is the health system [3].

Another model, the Rainbow Model by Dahlgren and Whitehead, proposes three possible levels: (1) depending on whether they are characteristics of individuals (micro); (2) depending on the context in which the interactions of different people are located (mezzo) in the case of the closest context, such as the family; (3) and depending on a more general context (macro), such as public policies [4]. There are other models, such as the Diderichsen model of social stratification and disease production and the Brunner, Marmot and Wilkinson's model of influences throughout life [5,6].

There are other models, such as Diderichsen's model that emphasizes the way societies are organized creates a gradient of social stratification and assigns people different social positions. The social position of individuals determines their health opportunities. And Brunner, Marmot and Wilkinson's model links social structure to health and disease through material, psychosocial, and behavioral pathways. Genetic, childhood, and cultural factors are important additional influences on population health

According to the WHO and the report by “Social determinants of health *The proven facts*,” referring to social determinants specifies that there are 10 topics that can influence health equity in various ways, which can be positive or negative [7,8]. Of these 10 topics, 4 stand out because of their close relationship in the health care system: social exclusion, work, unemployment, and social support. The records in the computerized clinical history of these 4 issues can be shown in the form of the following nonclinical diagnoses: difficulty in acculturation, stressful work schedule, unemployment, problems in living alone, and other specific problems related to the immediate environment.

Research shows that social determinants may be more important than medical care or lifestyle choices in influencing health [8-12]. Primary health care is essential for detecting and recording the SDHs in the community, since it is the main way for the population to access the health system [9,12].

The Spanish administration is promoting the recording of diagnoses in computerized clinical histories, with the aim of providing a benefit to the user, the professional, and the community [13,14]. Yet, practitioners tend to record, in most cases, only clinical diagnoses, despite evidence in the literature documenting that addressing social determinants can lead to improvements in health and reductions in social disparities in disease [15-17].

There are 2 major classifications of disease coding that are referenced worldwide. First, International Classification of Diseases (ICD-11) promoted by the WHO. The adaptation of this classification in the Spanish language is ”Clasificación Internacional de Enfermedades (CIE-11)” and the adaptation to Catalan language is “Classificació Internacional de Malalties (CIM-10).” This classification predominates in Spain because it is mandatory by law in hospital discharge reports; therefore, it is the classification used in the National Health Service both in primary care and hospital care [18-21]. Second, the International “Classification of Primary Care in the European Community” (ICPC-E) promoted by the World Organization of General Practitioners/Family. This classification is adapted to the Spanish language ”Clasifiación Internacional de la Atención Primaria Comité Internacional de Clasifiación de la Wonca” (CIAP-2). ICPC-E is the official classification system in primary care in countries such as Finland, Norway, and the Netherlands [22-25].

Spain is working with the electronic patient health record. Each region of Spain presents its own electronic program. Each region of Spain presents its program according to its area. In the region of Catalonia, the “ecap program” is used in the primary care setting, and this program covers 80% of the primary care centers in Catalonia. In the hospital setting, the most prevalent program is the “SAP program.” To unify the histories of the 2 areas, “HCCC program” is used, which unifies the tests, results, and follow-up of both areas. All these health programs are provided by the health department [25]. Although there are different health computer programs in the same region and in the different regions of Spain, all of them have the disease coding system in common since the Spanish legislation requires that hospital discharges be coded with the same coding system, which is the following: “ICD-11” of the WHO “this classification is adapted to Spanish by the WHO “CIE-11” [18-21,26].

Consequently, for the coding and recording of the SDHs in primary care consultations in Spain and Catalonia, “CIE-11” and “CIM-10” are used. Within this coding, there are “*Z codes*,” al chapter 24 at the CIE-11 and ICD-11, which are tools available to primary care practices for the measurement of the SDHs [18].

The benefits of having a good coding of social determinants in the software program of primary care centers are diverse [18,19,22,27]. On the part of primary care professionals, the recording of social determinants facilitates the recognition of the reason for consultation and optimizes consultation time. It also helps the continuity of care between professionals and services, optimizing treatments and tests, and promoting interventions based on the asset model [28,29]. In the asset model, care is based on the positive capability to identify problems and activate solutions, focusing on promoting healthgenic resources to promote self-esteem and coping skills of individuals and communities. This asset model is centered on health as opposed to the deficit model, which is centered on disease [30]. For users, it reduces the overdiagnosis of some social disadvantages that present in consultations and are coded with clinical diagnoses and the overtreatment of these with pharmacological remedies [31,32]. We define social disadvantages as the discomfort experienced by a person as a result of the social situation in which he or she finds himself or herself, such as a person visiting a health professional's office because he or she has insomnia as a result of being unemployed [32].

These users could benefit from a different approach to their clinical pathologies with community interventions or social prescriptions, which help people take greater control over their health, improve their social support network, and decrease the need for medicalization [32]. The present study aims to design and evaluate an intervention to increase the recording of SDHs in computerized medical records in primary care consultations in the area of Central Catalonia (Spain).

## Methods

### Study Design

Quasi-experimental, nonrandomized, controlled, multicentric study with 2 parallel study arms. The experimental group consists of those who have undertaken the intervention and labeled any of the 5 selected SDHs, and the control group includes those who have not received the intervention but have labeled any of the 5 SDHs.

### Data Collection and Sources of Information

This study was conducted in the area of Central Catalonia (Spain) with primary care professionals from the Institut Català de la Salut (ICS), who worked from September 23, 2019, to March 31, 2020.

The participation of the professionals was done by agreement. All professionals working in ICS at the Territorial Management of Central Catalonia (GTCC) were invited to participate on a voluntary basis, and all interested professionals were accepted.

Once the intervention was performed, monitoring was undertaken for those professionals who agreed to sign the informed consent form authorizing the researchers to monitor the number of 5 SDH entries recorded by the professionals over the following 6 months. Likewise, a control group was created, consisting of professionals who had labeled some of the 5 SDHs and had not performed the intervention. The categories that took part in the intervention were nursing, social work, medicine, and dentistry.

### Health Areas and Professionals Involved in the Study

A total of 22 basic health areas were available for recruitment in this study.

For the experimental group, 21 basic health areas accepted to participate and 74 professionals from these health areas accepted to participate. To enter the study, the interested professional had to complete the training and sign the informed consent. Once the informed consent was signed, the professional became part of the study in the experimental group. For the control group, 22 basic health zones were monitored. All the professionals worked with their work computer recording the SDHs selected in the study in the patient's computerized clinical history.

### Sample Calculation

The sample was a convenience selection, accepting all professionals interested in participating in the study. There were 934 professionals likely to participate in the study. We estimated that there would be 2 professionals for health care primary centers out of the 33 health care primary centers that would participate in the study, yet we made a sample calculation estimate.

A sample calculation was performed using the GRANMO [33].

The result was that a random sample of 72 individuals is sufficient to estimate, with a 95% CI and a precision of ±10 percentile units, a population percentage that is expected to be around 20%. The percentage of necessary replacements is expected to be 20%.

### Study Procedures

#### Selection of SDHs

A multidisciplinary working group was created consisting of a primary care nurse, a social worker, and a family and community physician. All of them have experience in community health and years of practice in the primary care setting. In order to make the selection, the vulnerability maps existing in the territory, the studies on SDHs, and the indicators proposed in these studies were reviewed and evaluated [17,34-39]. Subsequently, the work focused on the diagnoses of (ICD-10) Z55-Z65 ”*people with potential health risks related to their socio-economic and psychosocial situation*“ [18].

From this list, 5 nonclinical diagnoses were selected, each describing an SDH. The following criteria were used to select them: (1) relevance to the primary care practice as judged by experts in the working group; (2) relevance to community action in the primary care setting; (3) acceptability to primary care professionals and patients; and (4) consistency with socioeconomic indicators from different sources [7,35,38-40].

Finally, the SDHs selected were as follows: (1) unspecified unemployment; (2) stressful work schedule; (3) problems related to people living alone; (4) difficulty acculturating; and (5) other specific problems related to the immediate environment. We also worked with sociodemographic variables of the professionals, such as the category they represented and the work team.

#### Homogeneous Description

Once the 5 SDHs were selected, the variability in terms of their labeling was assessed. Consequently, a homogeneous description was made taking into account previous existing studies [18,19,21,39].

#### Intervention

In order to favor the recruitment of professionals in the study, a large-scale dissemination was carried out. First, it was presented to the management teams of the 3 primary care services (SAP) of the territorial management of Central Catalonia. Subsequently, it was presented to all the community health referents of the teams and the information was transmitted to the professionals.

This recruitment training was offered to the 3 primary care service areas and all professionals interested in participating in the study were accepted.

Community health referents aim to improve the health and well-being of the community and reduce social inequalities. They mobilize community resources, identify, and respond to the needs of the community and the social determinants exposed, and finally, empower community participation as agents in the process.

The intervention consisted of training professionals interested in participating in the study in order to learn about and increase the homogeneous registry of the 5 selected SDHs. Therefore, the intervention was carried out for all professionals interested in participating in the study.

Proportion of professionals who attended training: the number of professionals in the ICS in 2019 was 934 and the professionals who undertook the training were 147 professionals. The proportion of professionals who attended the training were 15.73% (n=147).

#### Criteria for Group Assignment

This is a nonprobabilistic sampling, where the experimental group consisted of professionals who undertook the training, signed the informed consent form, and registered any of the 5 SDHs selected in this study. And the control group consisted of professionals who did not take the training and who had registered any of the 5 SDHs selected in this study.

#### Characteristics of the Intervention

The intervention consisted of a 2-hour face-to-face training session. This training consisted of a theoretical and a practical part. In total, 9 training sessions were held on different days and in different areas in order to reach all professionals interested in participating.

The theoretical part consisted of explaining why and the methodology of the study, as well as the uniform definitions of the SDHs [18,19,39,41,42]. The practical part consisted of solving 18 practical cases that professionals could encounter in their primary care practice, and a checklist was used to minimize the time burden on health care providers in deciding whether to record or not. Subsequently, the professionals answered individually and corrected each other’s efforts, resolving doubts and reinforcing the theoretical part [41]. The answers provided by the participants were used by the study researchers to detect the weak points of the training and consequently to strengthen and adapt the support documents provided to the collaborating professionals on implementing the study.

Subsequently, the professionals who had completed the training and accepted the monitoring were offered further nonmandatory web-based training that consisted of solving 10 practical web-based cases; the corrections were commented on individually by the principal researcher, favoring feedback between the two. The experimental group was provided with supporting documents: a workflow and 2 videos, one of which was a summary of the training given in person and the other was a tutorial using the ”e-cap“ software program about when and how to record the selected 1 of these 5 social determinants of health.

The e-cap program is used by professionals in clinical and nonclinical data management of ICS primary care users.

**Figure 1:**
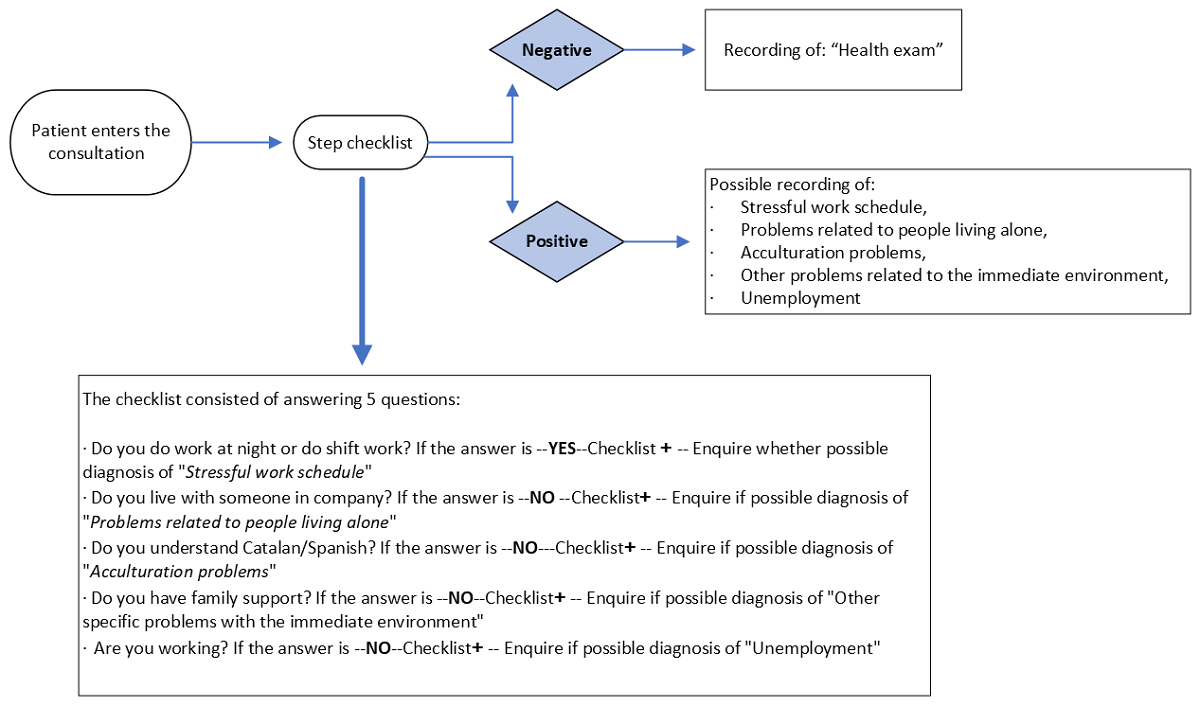
Experimental group workflow.

The following is a detailed description of the workflow of the experimental group (Figure 1).

The patient entered the primary care office and the health professional administered the checklist, which consisted of 5 questions where the patient answered “yes” or “no.”

If the result was “yes,” then it indicated the probability of recording the diagnosis of the social determinant to be treated.

The checklist was:

- Do you work at night? If the answer was ”YES“ the checklist was considered positive, then the practitioner was directed to the definition of the determinant ”Stressful work schedule.“ If it met the definition provided, the social determinant was recorded.
- Do you live with someone in company? If the answer was ”NO“ the checklist was considered positive, then the practitioner was directed to the definition of the determinant ”Problems in living alone.“ If you met the definition provided, the social determinant was recorded.
- Do you understand Catalan/Spanish? If the answer was NO the checklist was considered positive, then the practitioner was directed to the definition of the determinant ”Acculturation problems.“ If you met the definition provided, the social determinant was recorded.
- If the answer was NO the checklist was considered positive, then the practitioner was directed to the definition of the determinant ”Other specific with the immediate environment.“ If it the definition provided the social determinant was recorded.
- Are you working? If the answer was NO the checklist was considered positive, then the practitioner was directed to the definition of the determinant ”Unemployment.“ If the definition provided was recorded.

### Monitoring

The experimental and control groups were monitored for 6 months.

### Ethical Considerations

All the procedures used in this study were approved by the University Institute for Primary Care Research (IDIAP), Jordi Gol Health Care Ethics Committee (Code 19/079-P). The research was developed within the frameworks of the Helsinki Declaration and the Organic Law 3/2018, December 5, 2018, on Personal Data Protection and guarantee of digital rights. The information collected was anonymized, eliminating identifying characteristics, and attributing codes to the participants.

To ensure that professionals understood the study design and procedure, all of them in the intervention group had to read the study information form before signing the informed consent. It was detailed on what the study consisted of, the benefits, what they were asked to perform, the study's data protection regulations, and, finally, the name of the person to contact for any clarification, rectification, or withdrawal from the study.

All the participants in the intervention group signed the informed consent form, while the participants in the control group were not required to sign because a pseudoanonymization extraction was performed, where the data were anonymized prior to analysis. However, in order to perform the pseudoanonymization extraction, it was necessary the approbation from Jordi Gol i Gurina University Ethics Committee again. This described all the details of how it would be developed and specified the protection systems that would be used. This study did not have any type of economic compensation for the participants.

### Analysis and Modeling

The data were retrieved by the ICS Technical Area Service, and data extraction was formally requested after approval from the Ethics Committee.

Once the data extraction was performed, the sensitive data of the participants were anonymized by assigning codes to preserve privacy.

To guarantee its confidentiality, a file was created and located in a shared folder (Shared Windows, SMB) and on a server within the CPD of the Territorial Management of Central Catalonia. This server is not accessible from outside the sanitary ring network (it is not accessible from the internet). The CPD has the appropriate access, environmental and fire safety measures for this type of installation.

The server (FS-*GTCC-01) where the shared folder (AVIPRO) has daily, weekly, and monthly backup copies, as well as copies in other locations.

The folder where the file will be stored has restricted access to the principal investigator, and only she can view and edit the contents. The file will be password protected.

To retrieve the data from the database, we used the Oracle program, which was carried out by a professional who is part of the IT department of the territorial management of Central Catalonia. To analyze the data, the software “R” (R Foundation) was used and was carried out by a statistician from the research support unit of the Territorial Management of Central Catalonia.

### Statistical Analysis

Absolute frequencies and percentages for categorical variables, and the median with quartiles based on the distribution of the data for numerical variables were used for the descriptive statistics. To compare the 2 groups according to their differential characteristics in the baseline information, the Mann-Whitney test was used for numerical variables, and chi square or Fisher *F* test was used for categorical variables. To analyze the differences between preintervention and postintervention, the Wilcoxon test was used. Finally, a linear regression model was used to estimate the effect of the group on the total number of diagnoses adjusted for the professional category. All analyses considered a significance level of 5%, 95% CI and were analyzed with R statistical software (version 4.0.3; The R Foundation).

## Results

**Figure 2:**
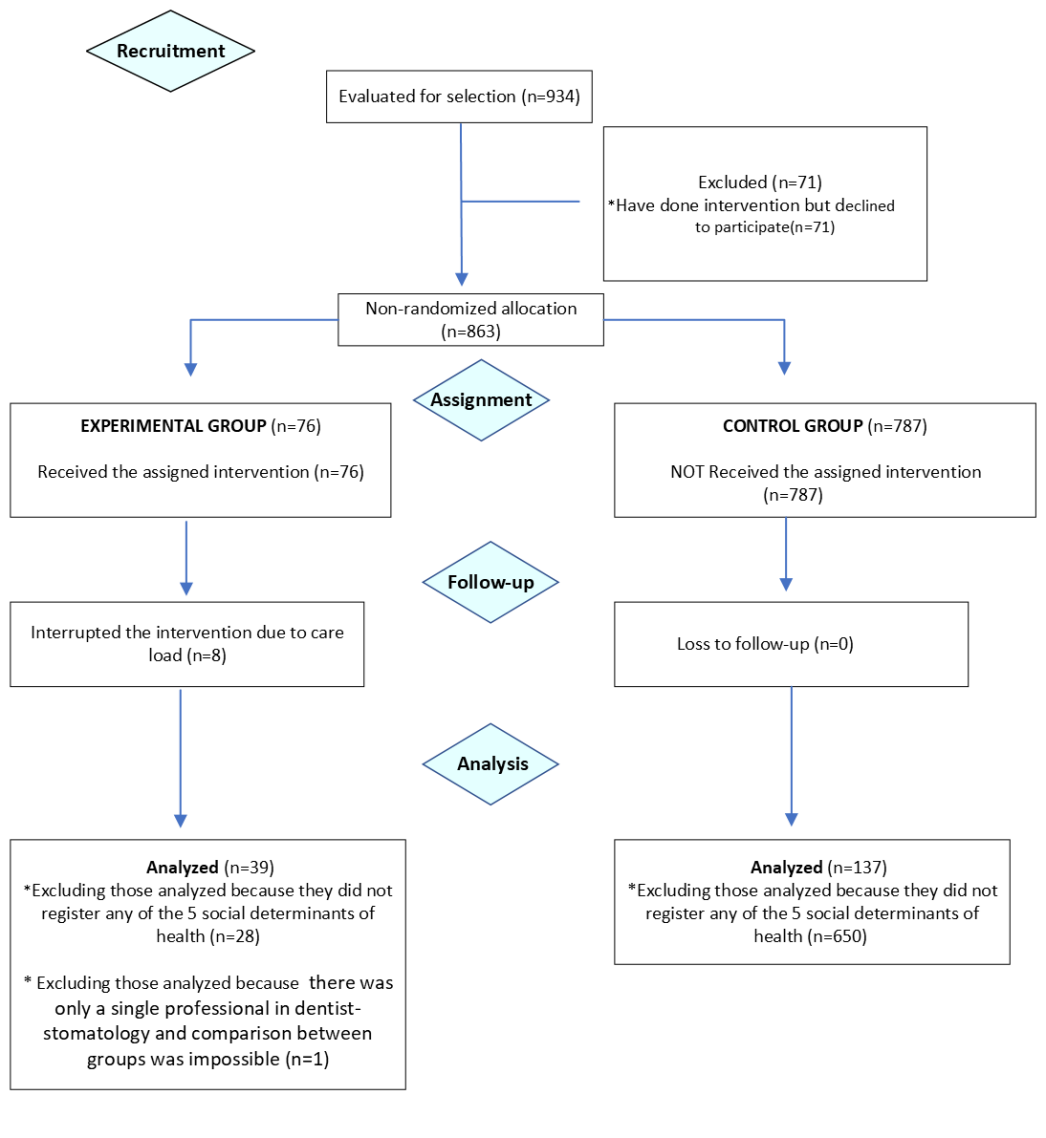
Consolidated Standards of Reporting Trials 2010 flow diagram.

Finally, there were 39 professionals in the experimental group and 137 in the control group as shown in Figure 2.

No significant differences were found in the study, neither by work area nor by category (Table 1).

In the analysis of results, the following category was discarded dentist-stomatologist because there was only a single professional in dentist-stomatology and comparison between groups was impossible. The category of medicine included: family medicine, family and community medicine, resident physician, and pediatric medicine.

As shown in Table 2, to assess the efficacy of the intervention, a comparison was made of the median number of diagnoses of the professionals who recorded any of the 5 SDHs in the experimental group compared with those in the control group.

In comparison to the experimental group, there was a significant increase in the median number of pre- and postintervention diagnoses (*P*<.001). This increase was significant in the nursing and medical categories, but not in the social work category, where a clear increase in diagnoses was observed. Even so, the sample was not large enough for the test to detect a difference as significant (Table 3). After the intervention, the professionals, in addition to increasing the number of times recorded, increased the diversity of labeling (Table 3).

**Figure 3:**
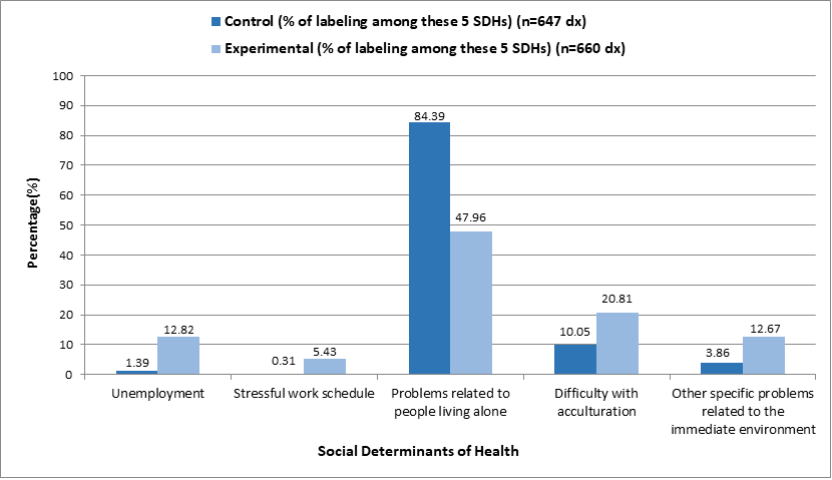
Description of the control group and the experimental group: distribution in the percentage of labeling of the 5 social determinants of health (SDHs).

**Figure 4:**
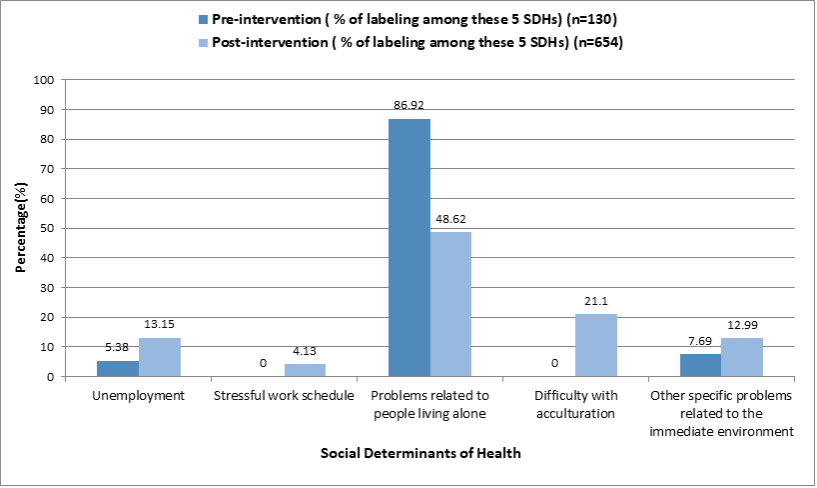
Description of the experimental group: distribution in the percentage of labeling of the 5 social determinants of health (SDHs).

Figures 3 and 4 show that prior to the intervention, professionals mostly labeled the diagnosis as “problems living alone.”

Using the linear regression model, the significant mean increase of the experimental group with respect to the control group was estimated with a coefficient of 8.18 (95% CI 5.11-11.26). There was a significant effect of the number of social work diagnoses with respect to the nursing category (12.93, 95% CI 9.42-16.43; Table 4).

**Table 1:** Description of the overall sample, control group, and experimental group.

Sample		Total professionals, n (%)	Control professionals, n (%)	Experimental professionals, n (%)	Comparison *P* values	
**Primary health care services**						.51
	Bages-Berguedà-Moianès	81 (46.03)	60 (43.79)	21 (53.85)		
	Anoia	51 (28.98)	42 (30.65)	9 (17.95)		
	Osona	44 (25.00)	35 (25.54)	9 (17.95)		
**Specialty**						.43
	Nurse	80 (45.45)	63 (45.98)	17 (43.59)		
	Family medicine	16 (9.09)	12 (8.75)	4 (10.25)		
	Family and community medicine	42 (23.86)	34 (24.82)	8 (20.51)		
	Resident physician	3 (1.70)	3 (2.19)	0 (0)		
	Dentist, stomatologist	1 (0.57)	0 (0)	1 (2.56)		
	Pediatrician	2 (1.14)	2 (1.46)	0 (0)		
	Social worker	32 (16.18)	23 (17.79)	9 (23.07)		

**Table 2:** Comparison of median log of the 5 social determinants of health recorded in the control group versus the experimental group.

Characteristics		Control, median (IQR)		Experimental, median (IQR)		Comparison^a^, *P* value
Total diagnoses		2 (1-4)		9 (3-23)		<.001
**By sector**						
	Nurse	2 (1-3)	4 (3-13)		<.001	
	Medicine	1 (1-1)	7.5 (1-10)		<.001	
	Social worker	9 (2.5-21.5)	28 (22-34)		.026	

^a^Mann-Whitney test.

**Table 3:** Comparison of median log of the 5 social determinants of health recorded from the experimental group before and after the intervention.

Characteristics		Preintervention, median (IQR)	Postintervention, median (IQR)	Comparison^a^, *P* value
Total diagnoses		0 (0-0)	8.5 (3-22.5)	<.001
**By category**				
	Nurse	0 (0-0)	4 (2.5-11.5)	<.001
	Medicine	0 (0-2)	8 (2-11)	.011
	Social worker	0 (0-24)	28 (22-34)	.164

^a^Wilcoxon test.

**Table 4:** Linear regression model. Reference categories: control group and nursing category.

		Coefficient	95% CI	*P* value
Intercept		3.17	(1.19 to 5.15)	.002
**Group**				
	Intervention	8.18	(5.11 to 11.26)	<.001
**Category**				
	Primary care physician	–1.7	(–4.48 to 1.15)	.24
	Social worker	12.93	(9.42 to 16.43)	<.001

## Discussion

### Principal Findings

Professionals who received the intervention labeled a higher median than those who had not, suggesting that the determinant existed but was not recorded. In the medical and nursing categories, there was more difference in coding, which could be attributed to lack of awareness of these ”Z codes“ as well as lack of clarity about who can document a patient's social needs [8,12,28,30,31,40]. With respect to the category of social worker, there was a smaller difference in the coding record because it is the category that deals with the social problems that are visible or requested by the patient.

A social worker is more familiar with this type of nonclinical diagnoses; however, an increase of recording in this category was also observed [17]. It should also be noted that the lack of homogeneous definition of nonclinical diagnoses describing the SDHs contributes to the difficulty or variability of recording [39].

The social determinant of ”problems in living alone“ requires a particular explanation when looking at the results. As shown in Figure 3, it can be seen that the control group has 84.39% (n=546) and experimental group has 47.96% (n=316.53). This determinant is the most coded in the both groups because it is a social determinant that administration has been promoting its recording due to the multifactorial problems it causes; therefore, for years, its recording has been encouraged in the primary care consultations of the ICS.

Comparing SDHs between the experimental group where diagnoses recorded during the 11 years prior to the intervention were also compared versus diagnoses recorded 6 months after the intervention, a significant difference (*P*<.001) can be observed. There is also a diversity of postintervention SDHs records, making the significance of the intervention visible [28].

A study was found in Canada where the lack of clinical records in primary care consultations was exposed. This study introduced a clinical diagnosis coding tool through a redesigned electronic medical record (EMR) interface. This tool was designed with physician support and integrated into the clinical workflow. The results of this study showed significant improvements in primary care coding and minimal disruption to routine clinical workflow [43]. In our opinion, this study makes us consider the implementation of this checklist explained in intervention in our study intervention to the computerized medical history program to help the health care professional in recording nonclinical diagnoses.

Another study describes the variability of records of social problems among health centers that serve populations with different social vulnerability. The same study demonstrated the low correlation between the deprivation index and Z-coding records in the primary care medical record, which could suggest undercoding of SDH records in primary care practices [37]. However, there are no official data for 4 of the 5 selected SDHs.

The involvement of experienced health care professionals in the conception and development of this intervention has been essential for an effective intervention that can be incorporated into the workflow of health care professionals. First, they were perfectly familiar with the functioning of the health record program, which allowed them to be aware of the advantages and disadvantages when planning the intervention. Second, they were knowledgeable about the health care practice encountered by the health care workers in their workflow as well as the workload presented.

The results obtained in the present research may have applicability in terms of favoring the implementation of SDHs recording using nonclinical diagnostics. This may be an element that contributes to the development of a professional practice based on healthgenic models, such as health assets. This could contribute to the reduction of overdiagnosis and pharmacological overtreatment in people with these SDHs.

Future research should continue to enhance and facilitate, through a workflow and a multiprofessional working group, the recording of these and other SDHs in the computerized medical records of primary care consultations.

The most relevant limitation of this study was that only 5 SDHs have been empowered to be recorded. However, there were other 5 SDHs that would have to be taken into account. Even so, studies such as the present one may encourage more and more SDHs to be recorded.

### Conclusions

The intervention described in the study, based on a checklist to encourage the recording of social determinants in the patient's medical record in primary care consultations, is an effective solution to increase the recording of social determinants in the computerized medical record.

In this sense, the intervention could be applied in the workflow of professionals. Promoting codification of determinants of health will improve both the population, through treatments with a healthgenic perspective and therapeutic appropriateness, and the professionals, optimizing resources, including time. It is necessary to continue promoting the recording of social determinants in primary care consultations by raising awareness of their recording and adapting the checklist to the records of the SDHs.

## Abbreviations

CIAP-2: Clasifiación Internacional de la Atención Primaria Comité Internacional de Clasifiación de la Wonca
CIE: Clasificación Internacional de Enfermedades
CIM: Classificació Internacional de Malalties
EMR: electronic medical record
GTCC: Territorial Management of Central Catalonia
ICD: Statistical Classification of Diseases and Related Health Problems
ICPC-E: Classification of Primary Care in the European Community
ICS: Institut Català de la Salut
SAP: health care service
SDH: social determinants of health
WHO: World Health Organization

## Data Availability

The data sets generated during this study are not publicly available because consent was not obtained from participants to share the research data publicly.
